# DFT Studies of the Activity and Reactivity of Limonene in Comparison with Selected Monoterpenes

**DOI:** 10.3390/molecules29071579

**Published:** 2024-04-01

**Authors:** Katarzyna Rydel-Ciszek

**Affiliations:** Department of Physical Chemistry, Faculty of Chemistry, Rzeszów University of Technology, Al. Powstańców Warszawy 6, 35-959 Rzeszów, Poland; kasiar@prz.edu.pl

**Keywords:** monoterpenes, global reactivity indices, limonene, cymene, pinene, thymol, menthol

## Abstract

Nowadays, the effective processing of natural monoterpenes that constitute renewable biomass found in post-production waste into products that are starting materials for the synthesis of valuable compounds is a way to ensure independence from non-renewable fossil fuels and can contribute to reducing global carbon dioxide emissions. The presented research aims to determine, based on DFT calculations, the activity and reactivity of limonene, an organic substrate used in previous preparative analyses, in comparison to selected monoterpenes such as cymene, pinene, thymol, and menthol. The influence of the solvent model was also checked, and the bonds most susceptible to reaction were determined in the examined compounds. With regard to E_HOMO_, it was found that limonene reacts more easily than cymene or menthol but with more difficultly than thymol and pienene. The analysis of the global chemical reactivity descriptors “locates” the reactivity of limonene in the middle of the studied monoterpenes. It was observed that, among the tested compounds, the most reactive compound is thymol, while the least reactive is menthol. The demonstrated results can be a reference point for experimental work carried out using the discussed compounds, to focus research on those with the highest reactivity.

## 1. Introduction

Limonene (1-isopropenyl-4-methylcyclohexene, C_10_H_16_) consists of two isoprene units and has two double bonds, one connecting carbon atoms in the ring, an internal or endocyclic bond, and the other occurring outside the ring, that is, external/exocyclic ones [1,2]. Limonene is a monoterpene that occurs naturally in citrus plants such as lemon, orange, and grapes, or even in olive oil [3] and has a wide range of applications. For example, limonene contained in orange peels can be used to produce biogas (using the leaching process) [4], or in the production of biodegradable materials (limonene then serves as an additive) [5]. It was shown that material enriched with a limonene–cyclodextrin/limonene inclusion complex added to poly(*L*-lactic acid) compared to poly(*L*-lactic acid) increases water permeability and absorption. This material has an increased barrier to UV–Vis light, which helps protect food against oxidation and also has antibacterial and antifungal properties [5]. The issue of cyclodextrin/limonene complexes is very developing. The incorporation of pure limonene into polymeric materials is difficult. The difficulties are caused by the temperature difference, since limonene has a low boiling point, while polymer processing reactions take place at much higher temperatures. However, the thermal stability of limonene can be increased by trapping it with the formation of an inclusion complex. The use of such a modification allowed the development of a method for obtaining linear polyethylene. Films containing polyethylene and the limonene–cyclodextrin inclusion complex have antifungal and, of course, antibacterial properties, so they can be used in the production of food storage packaging [6]. Interestingly, cyclodextrin and limonene complexes can also be used to improve the durability and aroma of soft drinks [7]. However, there are cases of biomass management in which the antibacterial properties of limonene are a significant disturbing factor; this applies to reactions in which bacteria are used. In the process of obtaining components for the production of biopolymers, using *Escherichia coli*, which used sugars from orange peel to obtain polyhydroxyalkanoates, limonene, due to its antibacterial properties, had to be eliminated using superoxides produced from potassium peroxide [8]. *Rhodococcus Globrulus* bacteria are also able to use components of eucalyptus oil, such as limonene, cymene, and thymol, as a source of carbon and energy. The identified and characterized bacterial cytochrome P450 type CYP108N12 is responsible for the biodegradation of these monoterpenes [9].

Limonene, cymene occur in light fractions of pyrolysis oil in car tires [10]. In order to separate these two monoterpenes, the tire pyrolysis oil epoxidation method before the separation was proposed. As a result of the reaction with hydrogen peroxide and a peroxophosphotungstate compound ([(C_18_H_37_)_2_N(CH_3_)_2_]_3_PW_4_O_20_), limonene undergoes epoxidation, while cymene does not react under these conditions. The resulting limonene epoxides can be successfully separated using a simple method—distillation, which also expands the possibilities of using waste materials [10].

In 2003, in accordance with the European directive (2003/15/EC), limonene was classified as an allergen [11]. Pure limonene has no allergic properties; however, in the presence of molecular oxygen in the form of long-term contact with air, its autoxidation is possible, and the limonene hydroperoxides formed as a result of this reaction are responsible for allergic reactions [12]. Hydroperoxides acting as an oxidant can oxidize the functional groups in proteins, for example, the sulfur residues of methionine and cysteine or the phenol group in tyrosine [13]. This is one of the reasons why essential oils, including, among others, limonene, cymene, and pinene, are unstable. It is known that limonene undergoes oxidation and degradation in an acidic environment. In order to limit this process, various stabilizers such as whey protein and an electrostatic whey protein–carboxymethyl cellulose complex were used [14]. Nevertheless, a change in the research environment and tests of toilet waters containing limonene showed its high stability [12]. During 9 months, there was no decrease in the initial concentration of limonene, suggesting that limonene is stable in typical water–alcoholic solutions. Unfortunately, a concentrated solution of limonene is much more easily oxidized and, under the influence of air, it may undergo autoxidation to form hydroperoxides [12]. On the other hand, limonene can also be used to deactivate free radicals [15].

**Scheme 1:**
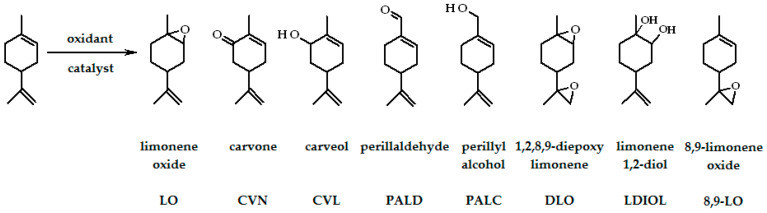
Possible products of limonene oxidation.

Limonene has found practical application in biorefineries, where post-production waste generated during the production of, e.g., orange juices, is processed into commercially useful compounds and constitutes a suitable raw material for the production of important products used in the flavor and fragrance industry [16] in the textile industry for the production of fibers [17] or in pharmacy/medicine [18,19]. However, limonene oxidation products are much more valuable than the substrate from which they are obtained and play an important role as an ingredient for the synthesis of fragrances or drugs [20,21], in the production of biodegradable polymers [22,23,24,25,26], or as a solvent/reactive diluent in the production of epoxy resins [26]. The main products of the C_10_H_16_ oxidation reactions are demonstrated in Scheme 1.

In the oxidation of limonene with molecular oxygen, it can undergo an autoxidation reaction, with the formation of limonene hydroperoxide as an intermediate product of the reaction [27]. Hydroxy- and alkylperoxide radicals can be abstracted to allylic hydrogens forming the ketone and alcohol as products, and the acylperoxyl radicals react with the double bond present in the alkene molecules, leading to the epoxide [28]. Bussi et al. [29] showed that the catalyst plays a crucial role in the initial stage of the reaction, consisting of the activation of the reactants and the decomposition of limonene peroxide with the formation of radicals. Nickel and aluminum hydrotalcites were used as catalysts for limonene oxidation by O_2_ conducted without additional solvent, resulting in the formation of epoxide, alcohol, and ketone [29]. Another heterogeneous complex tested in the oxidation of limonene with dioxygen carried out under mild conditions was the molybdenum (VI) catalyst MoCl_2_O_2_Bipy/TiO_2_-NT with 2,2′-bipyridine-4,4′-dicarboxylate (Bipy) ligand bounded to nanotubes (NT). In the photooxidation reaction (λ = 360 nm) catalyzed by the dioxo-type molybdenum complex, which proceeded practically without the participation of free radicals, limonene-1,2-oxide (LO) was the main product, but carvone (CVN) and carveol (CVL) were also observed [30]. Similar products were observed in another reaction with dioxygen–photooxidation (λ = 360 nm) using TiO_2_-NT dioxo-Mo (VI) complexes with ligands (*L*) such as Schiff base, bipyridine, terpyridine. The activity of Mo^VI^O_2_(*L*)/TiO_2_-NT depending on the ligand tested increases in order: Schiff base < bipyridine < terpyridine, where rich in electron ligands, act as “a bridge” for the electron transfer reaction [31]. In turn, by introducing another ligand of the type 2-aminothiazole-4-carboxylic acid and examining the photooxidation reaction of various monoterpenes, pinene was shown to be characterized by greater reactivity than limonene [32]. Using the complexes of iron(II)/(III) [33] and manganese(II) with 2,2′-bipyridine (bpy) [34] formed in situ in the presence of O_2_, in addition to epoxide, ketone, or alcohol, perillaldehyde and perillyl alcohole were also obtained. Limonene oxide can be selectively produced by the oxidation of limonene with dioxygen and bimetallic complex of ZnCo_2_O_4_, the reaction requires isobutyraldehyde as a mediator [35], as well as the use of silylated TiO_2_ P25 and solar radiation [36].

Oxidants, such as hydrogen peroxide or *t*-butyl hydroperoxide, are also applied in the oxidation reaction of limonene, and their use was tested in the case of using limonene both as a solvent—the reaction medium and as its substrate, similarly to the use of dioxygen [29]. To ensure hydrophobicity between the limonene as organic solvent and the aqueous phase associated with the addition of an oxidant, the complexes [MoO_2_(SA(T)P)]_2_, [MoO_2_(SATP)]_2_ [SA(T)P–salicylideneamino(thio)phenolate] for *t*-Bu-OOH [37] or Ti-salicyldimine with octadecyltrimethoxysilane for HOOH [38] were examined. The data summarized in Table 1 also provide information regarding the use of other oxidants in the limonene oxidation process. Compared to products obtained using dioxygen as an oxidant, diepoxide (DLO), 8,9-LO, or polymer were additionally formed. For HOOH, the following complexes were used: cobalt sandwich-type polyoxometalates [39], tungstophosphates [2], polyoxotungstates [11], Schiff base complexes with Co(II) and Cu(II) and the same compounds but immobilized in zeolite-Y [40], manganese(II) acetylacetonate on MCM41 [41], Al_2_O_3_ [42], the ions of non-transition metal [1], methyltrioxorhenium with different ligands [43], activated carbon where the active phase was the magnetite Fe_3_O_4_ [44] or MoO_2_ [45], complexes of VO and copper(II) with Schiff base ligands entrapped in the supercages of zeolite-Y [46], homogeneous and heterogeneous VO and iron(II) with Schiff base ligands [47], γ-Fe_2_O_3_/SiO_2_-NHFeP prepared from nanospheres and 5,10,15,20-tetrakis (pentafluorphenylporphyrin) iron(III) [48], heterogeneous Mn(III), Fe(III), and Co(III) porphyrin-based complexes immobilized on zeolite [49] or others complexes based on zeolite-Y [50,51,52] in which enclosing the catalyst in the porous structure of the support prevents the dimerization of the complexes, ensuring their catalytic activity. Catalysts used with *t*-butyl hydroperoxide as the oxidant are also zeolites, e.g., zeolite-Y with entrapped VO with Schiff base ligands [50], organic hybrid materials [26,53], Ti-MCM-41, and Ti-MWW compounds [54], iron(II) [55], molybdenum(II) complexes [56], salen-like Jacobsen’s catalysts with manganese(III) [57] or carbon-based complexes with cobalt(II) acetylacetonate [58]. Jacobsen’s compounds with manganese(III) [59,60] with Mn(II), Ni(II), Co(II) [61], or VO(Salten) anchored on SBA-15 (Salten–3-[*N*,*N*′-bis-3(salicylidenamino)ethyltriamine]) [62] were also used with other oxidants such as KHSO_5_ (used as ozone), iodosylbenzene, sodium hypochlorite, or urea hydroperoxide.

Interestingly, some of the mentioned catalysts are also used in the oxidation of other monoterpenes. For example, using analogous catalysts, oxidants, and solvents as given in Table 1, the pinene oxidation reaction was carried out for the conditions presented in Table 1, entries: 1 [30], 2 [32], 16 [40], 23 [47], 25 [52], 26 [51], and the products of these reactions were mainly ketone (verbenone) and alcohol (verbenol). Similarly, the review monograph on biomass management focusses, among others, on the use of both limonene and pinene [64]. In the literature review presented above, in addition to limonene, pinene [14], cymene [9,10], and thymol [9] were mentioned. These natural compounds, similar to limonene, are obtained from plants [65,66,67] and are monoterpenes known for their unique aromatic, therapeutic properties [65,67,68,69]. In turn, due to structural similarity, thymol is often discussed with menthol [70,71,72], a monoterpene resembling hydrogenated thymol, found, e.g., in mint [73], which also has numerous applications [74,75,76]. Interestingly, menthol can be formed from limonene through enzymatic reactions during the monoterpene biosynthetic pathway in peppermint [77]. Therefore, it seems interesting to conduct research on a larger group of monoterpenes, trying to correlate their theoretical activity with data obtained from experiments. In contrast to homogeneous catalysts, their heterogeneous counterparts are recovered, which makes them much more gentle on the environment [47]. Some heterogeneous catalysts are rinsed with large amounts of water and acetone after each catalytic cycle for reuse [46]. Rinsing with water is also carried out to remove undesirable ions, like chloride, and this reaction may contribute to coordinated H_2_O molecules in space, e.g., zeolite. Furthermore, it has been shown that such complexes (with coordinated water molecules) provide a higher conversion of limonene [46]. On the other hand, the presence of water affects the products of the limonene oxidation reaction and contributes to the ring opening reactions, as a result of which a diol (LDIOL) is obtained from epoxide (LO) [37]. Thus, it seems advisable to investigate the influence of solvents, including water, on the activity of monoterpenes. This can be performed using computational chemistry methods assuming appropriate PMC solvent models. Additionally, although the thesis, that all reactions can be carried out by selecting appropriate catalysts and “additives” as co-catalysts, presented in the review is true [78]. However, in terms of planning preparatory research and selecting appropriate substrates for various reactions, it is crucial to determine their activity in order to exclude the least reactive compounds in the initial stage of the research, which was used, for example, to separate a mixture of oils [10].

For this reason, using DFT computational methods, monoterpenes were examined to determine their activity and to check the possibility of a potential attack of selected monoterpenes on the empty orbital of the catalyst, which is consistent with the Dewar–Chatt–Duncanson model [30]. Monoterpenes were selected based on the review of the literature presented in the manuscript as compounds that can occur in oils in the presence of limonene, the substrate that is the object of my interest and previous research [79]. Determining the differences in the reactivity of analyzed compounds has many applications; for example, it may contribute to the separation of oil components from their mixture, as was achieved in [10], in which the limonene oxidation product (LO) was successfully separated from cymene by distillation. The influence of the use of the solvent model on selected monoterpenes was also examined, and their bonds that are most susceptible to breaking were determined.

## 2. Results and Discussion

**Scheme 2:**
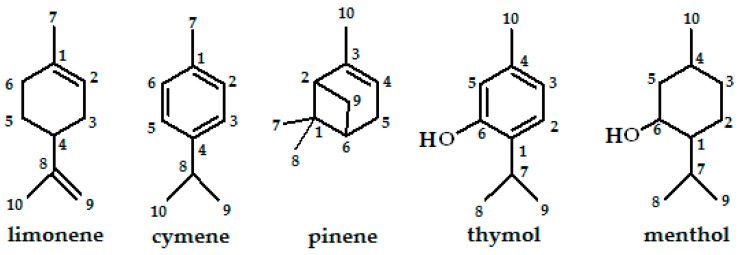
Structures of selected monoterpenes.

Limonene and the structures of selected monoterpenes such as cymene, pinene, thymol, and menthol were optimized using methods with different hybrid functionals and basis sets, Table S1. However, for the tested compounds, the best correlation with experimental data [10] was provided by the B3LYP/6-311+G(d) level method; therefore, it was used to calculate reactivity descriptors. These structures, along with the numbering of carbon atoms presented in GaussView03 and used to discuss BDE, are shown in Scheme 2.

For the optimal structures of selected monoterpenes generated after the calculations performed in the gaseous phase, calculations were also conducted, assuming that the PCM solvent model works for solvents with different polarities, such as water, acetonitrile, and methanol. For each optimized structure, the energy of the highest occupied molecular orbital (HOMO) and the lowest unoccupied molecular orbital (LUMO) was generated, and these data along with the E_gap_ value are presented in Table 2. Those frontier orbitals are the most important orbitals in terms of reactivity [80]. The HOMO energy determines the molecule’s susceptibility to electrophilic attack and is related to the ionization potential. Analogously, the LUMO energy determines the molecule’s predisposition to nucleophilic attack and is related to electron affinity [81]. In turn, the lower the E_gap_, the less energy is needed to transfer an electron from the HOMO orbital to the LUMO [80]. Therefore, knowing the difference between the HOMO–LUMO energy, it is also possible to determine which of the tested molecules is characterized by the greatest kinetic stability [82].

**Figure 1:**
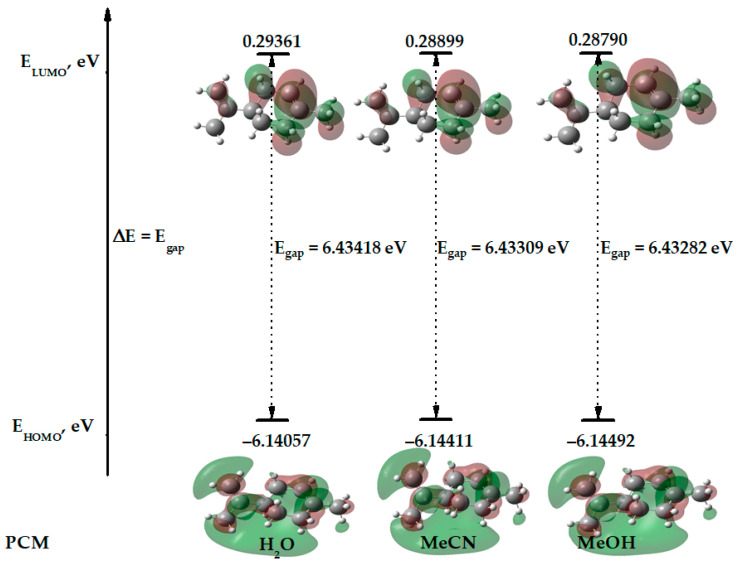
Energies of the HOMO and LUMO molecular orbitals for the limonene molecule with their visualized surfaces for the corresponding orbitals, B3LYP/6-311+G, assuming different solvent models.

Analyzing the E_gap_ values collected in Table 2, it was found that, regardless of the solvent model used, the most reactive monoterpene is thymol, which is characterized by the lowest stability, while menthol will be the least reactive of the group of compounds tested. It was also observed that, depending on the tested monoterpene, E_gap_ (E_gap_ = E_LUMO_ − E_HOMO_ [83,84]) can be used to determine the dependency on the solvent model used. For example, on the basis of the data collected in Figure 1, which visualize the surfaces of the HOMO, the LUMO molecular orbitals with their corresponding energies for different ε for limonene, it can be observed that the HOMO–LUMO energy difference increases with increasing ε and is the largest for water, then acetonitrile, and then methanol.

However, analyzing the data collected in Table 2 for the other monoterpene molecules requires individual analysis. Pinene, like limonene, has the largest E_gap_ for using water as a solvent model and the smallest without assuming a solvent model, while the difference between the E_LUMO_ and E_HOMO_ orbitals for cymene for water as a solvent model is the smallest. However, when using the solvent model, remember that it is a polarized continuum model in which solvents are represented by the dielectric continuum medium, and therefore the analyzed models should be verified by conducting experimental work.

**Figure 2:**
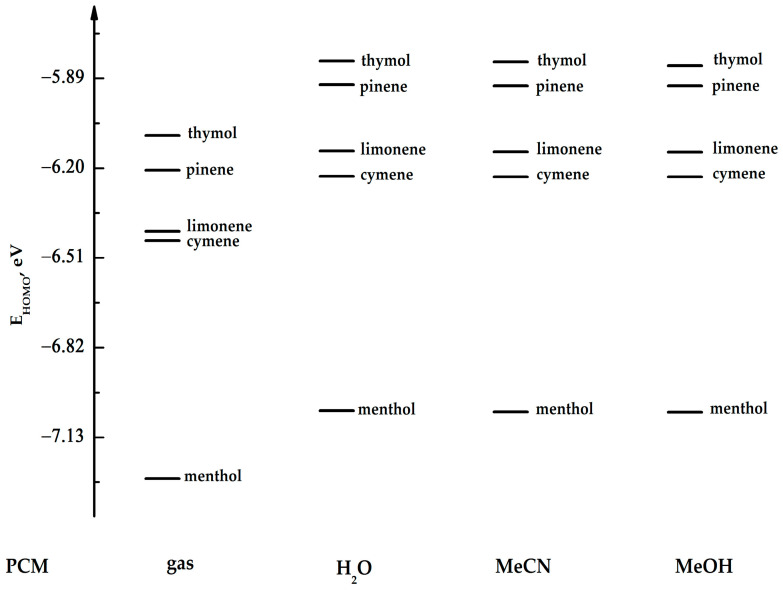
Energies of the HOMO molecular orbitals for limonene, cymene, pinene, thymol, and menthol, B3LYP/6-311+G, assuming different solvent models.

Nevertheless, of the ε value, the HOMO energy values of the tested monoterpenes can be arranged as follows: thymol > pienene > limonene > cymene > menthol, as shown in Figure 2, thymol is the compound with the highest E_HOMO_ (in all PCM models), while menthol, among the selected monoterpenes, is the compound with the lowest value of this energy.

In the reaction of monoterpenes with an oxygen-activated catalyst, in the case of electron-rich olefins, the oxygen atom transfer step is based, according to the Dewar–Chatt–Duncanson model, on the attack of the (nucleophilic) olefin on the empty LUMO orbital of the oxygen-catalyst [30]. Therefore, having the values of the highest occupied molecular orbitals, it can be concluded that the monoterpene with the highest E_HOMO_ will be the most reactive in atom transfer reactions. Based on Figure 2, it follows that thymol and pinene are more reactive than limonene, while cymene and menthol are less reactive. The presented results are consistent with previously presented literature data that showed that pinene was characterized by greater reactivity than limonene [32], while from another article, it follows that cymene compared to limonene does not react [10].

The energy of HOMO and LUMO is used to determine the global chemical reactivity descriptors (GCRD)—Table 3. GCRDs are defined for monoterpene molecules in their singlet ground state with the DFT of Parr, Pearson, and Yang [85]. The ionization potential (I), correlated with –E_HOMO_, is the minimum energy that is necessary to remove an electron from a monoterpene molecule [84]. In all the cases listed in Table 3, thymol has the lowest ionization potential value, followed by pinene, limonene, and cymene. Menthol has the highest ionization potential. In turn, the electron affinity (A) is determined on the basis of the value of –E_LUMO_ and characterizes the ability to attach an electron, resulting in the formation of a negative ion. Table 3 shows that, for the results of calculations carried out without assuming the solvent model, thymol has the highest A value. The situation is similar when using MeOH as a PCM model—thymol is also characterized by the highest electron affinity value of all the tested compounds. In turn, menthol has the lowest A value in all the tested solvent models. The electronegativity (X) values, i.e., the measure of the tendency to “attract” electrons [84], is defined as X = 0.5∙(I + A) [86,87,88] and increase in series: pinene, limonene, thymol, cymene, and menthol with the highest value. Global hardness (η) according to Parr and Pearson—first-order derivative of the chemical potential with respect to the total number of electrons N, with a constant external potential, or second-order derivative of energy (also with respect to the number of electrons N, at a constant external potential). The global hardness is calculated on the basis of the knowledge of I and A. Global softness (S) is related to η. Global hardness and softness concern the sensitivity of electron–electron interactions; for example, for anions that are characterized by the lowest hardness value and the highest softness, their susceptibility to changing the number of electrons is low [89]. For the analyzed monoterpenes, molecular hardness η = 0.5∙(I – A) and molecular softness S = 0.5/η [86,87,88] were calculated; the η values increase from thymol (with the lowest η value), cymene, pienene, limonene, and menthol (with the highest η value and the lowest S value). The global softness values in the given series decrease. In Table 3, ω (where ω = μ^2^/(2∙η) [88,90]) is characterized by the electrophilicity index, which describes the global electrophilic nature of molecules. It expresses the measure of energy reduction resulting from the flow of electrons between a donor and an acceptor. In the case of a reaction, a molecule with a higher ω will react as an electrophile, while a molecule with a lower ω—as a nucleophile [88]. For example, using water as the PCM model, menthol has high values of both the ω and the X descriptor, so it can act as an electrophile. The last column of Table 3 applies to μ, which describes the chemical potential; μ = −0.5∙(I + A) [90]. Chemical potential shows the sensitivity of the system to changes in electrons, a high μ indicates that the molecule has the properties of a strong electron acceptor, while a low μ characterizes strong electron donors [84]. Of the compounds analyzed, menthol has the lowest μ value; a low μ and high ω for a molecule indicate its good electrophilic nature.

Based on the data collected in Table 3, it can be concluded that, in light of the theory of hard and soft acids and bases (HSAB), thymol should react the easiest/fastest because of its lowest η value, which proves its nucleophilic properties and proton acceptor capabilities, while menthol is the least reactive of the tested group of compounds.

Furthermore, the dissociation enthalpies (BDEs) of individual C–H in the molecules of the tested monoterpenes were calculated according to the reaction:

BDE = *H*_monoterpene without-H_ + *H*_Hatom_ − *H*_monoterpene_.

The energies required for homolytic breakage of a specific bond calculated using two calculation methods are presented in Tables S2–S6. The numbering of carbon atoms in monoterpene molecules is consistent with the numbering shown in Scheme 2. The data in Tables S2–S6 are listed from the lowest BDE values, i.e., from the bonds in monoterpene molecules that are easiest to break. When comparing the results of the BDE calculations, it can be concluded that, regardless of the chosen calculation method, they provide consistent results. The lowest energy needed to remove a hydrogen atom from the analyzed molecules (rounded to whole numbers) is 81 kcal/mol for the limonene molecule, 77 kcal/mol for thymol, 74 kcal/mol for pinene, 83 kcal/mol for cymene, and 91 kcal/mol for menthol. Therefore, pinene and thymol can most easily undergo reactions involving the transfer of a hydrogen atom to the catalyst molecule, while menthol from the tested group of compounds is the most difficult to oxidize. Data from Tables S2 and S4 show that, for limonene and pinene, the oxidations should occur more easily in the allylic position, which is consistent with the literature data [47]. Additionally, this experimental work confirms that pinene is more reactive than limonene, and one of the main products resulting from the oxidation of both limonene and pinene under similar conditions are their allylic derivatives (Table 1, entries: 16 [40], 23 [47], 25 [52], 26 [51]). In turn, in the case of cymene and limonene and using the same reaction conditions, these monoterpenes were found to oxidize at carbon C7 [9,91]. Interestingly, in the case of both monoterpenes, the calculated BDE values for these bonds are comparable (Tables S2 and S3). In the case of BDE calculated for cymene (Table S3, Scheme S1), the C8-H bond is the easiest to break, which is also confirmed by the products obtained in preparative experiments [92]. In thymol and menthol molecules, the breaking of bonds leading to the formation of the corresponding ketones (Tables S5 and S6, Scheme S1) is favored [77,93,94,95].

## 3. Materials and Methods

Calculations of thermodynamic parameters were performed in Gaussian 09 and 16 [96] using DFT methods with the Becke 3-parameter hybrid density functional, Lee Young Parr correlation B3LYP [97] and the basis sets 6-31g(d), 6-311+G, Def2SVP, or functionals CAM-B3LYP; B3PW91; ωB97XD [98,99] with 6-311+G(d). The GaussView03 programme was used to model the structures of monoterpenes molecules. Geometry optimization was performed using the B3LYP/6-31G(d), 6-311+G(d) level or Def2SVP method [100,101] using as PCM model water (ε = 78.3553), acetonitrile (ε = 35.688), and methanol (ε = 32.613). The values reported in this paper, combining electronic energies with the enthalpy correction, were used for bond dissociation energy (BDE) calculations. BDE is expressed as a change in the enthalpy of the homolytic cleavage of a selected bond [102] and is one of the basic features of the reactivity of selected compounds.

## 4. Conclusions

The presented results can contribute to the rational planning and optimization of the experimental work. On the basis of the calculations of the ionization potential, electron affinity, global hardness and softness, the electrophilicity index, electronegativity, and chemical potential, it was found that of the analyzed monoterpenes, thymol will be more reactive than limonene. The most stable and least reactive is menthol, which means that its presence, among others, in essential oils is least exposed to subsequent reactions. Additionally, the energy values of the highest occupied molecular orbital show that E_HOMO_ limonene is “in the middle” of the tested monoterpenes and allow for the ranking of the examined compounds from the most reactive ones, respectively: thymol, pienene, limonene, cymene, and menthol. These calculations are partially confirmed by experimental data [14,32]. The use of the solvent model does not significantly affect the structures of the analyzed monoterpenes. The calculated values of bond dissociation enthalpy also confirm the conclusions obtained from the GCRD analysis that pinene and thymol undergo hydrogen atom transfer reactions more easily than limonene.

## Figures and Tables

**Table 1:** Complexes, oxidants, solvents and main oxidation products used in limonene oxidation reactions.

Entry	Catalysts	Oxidants	Solvents	Oxidations Products	Ref.
1	MoCl_2_O_2_(Bipy)/TiO_2_-NT	O_2_/(λ = 360 nm)	MeCN	LO, DLO, CVN	[30]
2	MoO_2_(*L*_1_–*L*_4_)/TiO_2_-NT	O_2_/(λ = 360 nm)	MeCN	LO, DLO, CVN	[31,32]
3	CYP108N12	O_2_	Tris-HCl	PALC, PALD	[9]
4	[Fe(bpy)_2_]^2+^, [Fe(bpy)_2_]^3+^	air, O_2_	MeCN	LO, CVN, CVL, PALD	[33]
5	[Mn(bpy)_2_]^2+^	air, O_2_	MeCN	LO, CVN, CVL, PALD, PALC	[34]
6	TiO_2_-P25	O_2_	MeCN	LO	[36]
7	ZnCo_2_O_4_, isobutyraldehyde	O_2_	MeCN	LO	[35]
8	Pd(OAc)_2_/PTSA/BQ, Na_2_PdCl_4_/PTSA/BQ	O_2_	MeOH, ethanol, 2-Propanol	allylic ethers	[63]
9	NiAl-HT	O_2_	Limonene	LO, CVN, CVL	[29]
10	[MoO_2_(SAP)]_2_, [MoO_2_(SATP)]_2_	*t*-Bu-OOH	Limonene	LO, LDIOL	[37]
11	Ti:OTMS	H_2_O_2_	Limonene	CVN, CVL, LO	[38]
12	Na_10_[Co_5_W_19_O_70_H_4_]·44H_2_O	air, H_2_O_2_	MeCN, MeOH, acetone	LO, CVN, CVL	[39]
13	[M_4_(H_2_O)_2_(PW_9_O_34_)_2_]*^n^*^−^, M–Co^II^, Mn^II^, Fe^III^	H_2_O_2_	MeCN	LDIOL, CVN, CVL	[2]
14	[(C_18_H_37_)_2_N(CH_3_)_2_]_3_PW_4_O_20_,	H_2_O_2_	Tire pyrolysis oil	LO, DLO, LDIOL	[10]
15	(*n*Bu_4_N)_3_[NaHAsW_9_O_33_[P(O)R]_2_] (R = *t*-Bu or CH_2_CH_2_COOH) (*n*-Bu_4_N)_3-_[NaHPW_9_O_34_[As(O)*p*-C_6_H_4_NH_2_]_2_]	H_2_O_2_	MeCN	LO, DLO, LDIOL	[11]
16	Co(II)-Y, Cu(II)-Y with Schiff base ligands	H_2_O_2_	MeCN	CVN, CVL, LO, LDIOL	[40]
17	[Mn(acac)_2_APTS]@MCM-41	H_2_O_2_	Acetone–*t*-butanol	LO, CVL, CVN, polymer	[41]
18	Al_2_O_3_	H_2_O_2_	Ethyl acetate	LO, DLO, 8,9-LO	[42]
19	Ga(NO_3_)_3_, Al(NO_3_)_3_	H_2_O_2_	Ethyl acetate, THF	LO, DLO, LDIOL, 8,9-LO	[1]
20	MTO:L_5-7_	H_2_O_2_	CH_2_Cl_2_	LO, 8,9-LO, DLO, CVL, CVN	[43]
21	carbon EuroPh with Fe	H_2_O_2_	MeOH	PALC, CVL, CVN, LO, LDIOL	[44]
22	[VO(*L*_8_)H_2_O]-Y, [Cu(*L*_8_)H_2_O]-Y	H_2_O_2_	MeCN	LDIOL, CVL, CVN, LO	[46]
23	[VO(sal_2_bz)]_2_, [VO(sal_2_bz)]_2_-Y [Fe(sal_2_bz)(H_2_O)_2_]_2_⋅2H_2_O, [Fe(sal_2_bz)(H_2_O)_2_]_2_-Y	H_2_O_2_	MeCN	LDIOL, PALC, CVN, CVL	[47]
24	Mn(III)/Fe(III)/Co(III)/*L*_9_/Y/ammonium acetate	H_2_O_2_	MeCN	LO, 8,9-LO	[49]
25	[Fe^II^(*L*_10_)_2_(H_2_O)_2_]-Y	H_2_O_2_	MeCN	CVN, CVL	[52]
26	RuY, FeY, 3Y–6Y	H_2_O_2_	MeCN	CVN, CVL, LO, LDIOL	[51]
27	γ-Fe_2_O_3_/SiO_2_-NHFeP	*m*-CPBA, H_2_O_2_	MeCN	LO, CVN, CVL	[48]
28	MoO_2_-EuroPh	H_2_O_2_, *t*-Bu-OOH	MeOH	CVN, CVL, LO, PALC	[45]
29	[VO(VFCH)_2_]-Y, [VO(VTCH)_2_]-Y, [VO(SFCH)·H_2_O]-Y, [VO(STCH)·H_2_O]-Y	H_2_O_2_, *t*-Bu-OOH	MeCN	LO, LDIOL, CVN, CVL	[50]
30	[MoO_3_(Hpto)]∙H_2_O	*t*-Bu-OOH	α,α,α- trifluorotoluene	LO, LDIOL, DLO	[53]
31	[MoO_3_(Hpytz)]	*t*-Bu-OOH		LO, LDIOL, DLO	[26]
32	Ti-MCM-41, Ti-MWW	*t*-Bu-OOH	MeOH	LO, CVN, CVL, PALC	[54]
33	FePcCl_16_-NH_2_-SiO_2_	*t*-Bu-OOH	Acetone	CVN, LO, CVL	[55]
34	cobalt(II)-(acac)-carbon-based catalysts	*t*-Bu-OOH	Acetone–*t*-butanol	LO, CVN, CVL, polymer	[58]
35	(η^5^-C_9_H_7_)Mo(CO)_3_Me	*t*-Bu-OOH	Decane, *t*-butanol	LO, DLO, LDIOL	[56]
36	MCM-41-Mn(4-OHsalen), MCM-41Mn(4-OHsalhd), MCM-41 Mn(4-OHsalophen)	*t*-Bu-OOH	Acetone–*t*-butanol	LO, CVN, CVL, polymer	[57]
37	Mn(III)-Jacobsen-type catalysts	KHSO_5_	Acetone–H_2_O	DLO	[60]
38	Mn(Salen)Cl∙H_2_O	PhIO	CH_2_Cl_2_	LO, CVN, PALD	[59]
39	M(Salen)Cl∙H_2_O M = Mn^II^, Ni^II^, Co^II^	PhIO, NaOCl	Acetone, MeCN, CH_2_Cl_2_, ethyl acetate	LO, CVN, CVL	[61]
40	VO(Salten)-SBA-15	UHP	MeCN	LO, CVN, CVL, carvacrol	[62]

Abbreviations: Oxidants: *m*-CPBA—3-chloro peroxybenzoic acid; *t*-Bu-OOH—*t*-butyl hydroperoxide; PhIO—iodosylbenzene; UHP—urea hydroperoxide. Solvents: THF—tetrahydrofurane. Ligands: NT—nanotube; Bipy—2,2′-bipyridine-4,4′-dicarboxylate; *L*_1_—Schiff base; *L*_2_—bipyridine; *L*_3_—terpyridine; *L*_4_—2-aminothiazole-4-carboxylic acid; bpy—2,2′-bipyridine; PTSA—*p*-toluenesulfonic acid; BQ—benzoquinone; HT—hydrotalcites; SA(T)P—salicylidene amino(thio)phenolate; OTMS—octadecyltrimethoxysilane; ATPS—aminopropyltrimethoxysilane; MTO—methyltrioxorhenium; *L*_5_—*t*-butylpyridine; *L*_6_—4,4′-dimethyl-2,2′-bipyridine; *L*_7_—pyrazole; *L*_8_—(Z)-4-(((2hydroxyphenyl)amino)methylene)- 3-methyl-1-phenyl-1H-pyrazol-5-ol; sal_2_bzH_2_—(Z)-2-[4′-Methyleneamino-biphenyl-4-ylimino)- methyl]-phenol; *L*_9_—5,10,15,20-tetra(4-methoxyphenyl) porphyrin; *L*_10_—(*Z*)-2-((4-hydroxybenzylidene)-amino)benzoic acid; FeY—Na_26.1_Fe_1.3_[(AlO_2_)_30_(SiO_2_)_162_; RuY—Na_27.3_Ru_0.9_[(AlO_2_)_30_(SiO_2_)_162_; 3Y—Na_27.4_[Fe_0.86_(1)_0.61_(AlO_2_)_30_(SiO_2_)_162_; 4Y—Na_27.12_[Fe_0.96_(2)_0.59_(AlO_2_)_30_(SiO_2_)_162_; 5Y—Na_28.41_[Ru_0.53_(1)_0.46_(AlO_2_)_30_(SiO_2_)_162_; 6Y—Na_28.62_[Ru_0.46_(2)_0.41_(AlO_2_)_30_(SiO_2_)_162_; VTCH—vanillin thiophene-2-carboxylic hydrazine; VFCH—vanillin furoic-2-carboxylic hydrazone; H_2_STCH—salicylaldehyde thiophene-2-carboxylichydrazone; H_2_SFCH—salicylaldehyde furoic-2-carboxylic hydrazone; Hpto—5-(2-pyridyl-1-oxide) tetrazole; Hpytz—5-(2-pyridyl)tetrazole; Pc—phthalocyanine; 4-HOsalen)—N′-bis(4-hydroxysalicylaldehyde) ethylenediamine; 4-HOsalhd—N,N’-bis(4-hydroxysalicylaldehyde) cyclohexane-diimine; 4-HOsalophen—N,N′-bis(4-hydroxysalicylaldehyde)phenylenediamine; Salten—3-[*N*,*N*′-bis-3(salicylidenamino)ethyltriamine.

**Table 2:** Energy of the HOMO and LUMO orbitals along with ΔE_gap_ levels for limonene and selected monoterpenes, structures optimized using the method without and with PCM model.

	Gas			H_2_O			MeCN			MeOH		
	E_HOMO_, [eV]	E_LUMO_, [eV]	E_gap_, [eV]	E_HOMO_, [eV]	E_LUMO_, [eV]	E_gap_, [eV]	E_HOMO_, [eV]	E_LUMO_, [eV]	E_gap_, [eV]	E_HOMO_, [eV]	E_LUMO_, [eV]	E_gap_, [eV]
limonene	−6.41867	−0.02367	6.39500	−6.14057	0.29361	6.43418	−6.14411	0.28890	6.43309	−6.14492	0.28790	6.43282
cymene	−6.45105	−0.32817	6.12288	−6.22846	−0.11647	6.11199	−6.23010	−0.11783	6.11227	−6.23037	−0.11810	6.11227
pinene	−6.20724	0.02748	6.23472	−5.91145	0.33198	6.24343	−5.91553	0.32708	6.24261	−5.91635	0.32627	6.24262
thymol	−6.08669	−0.39212	5.69457	−5.83036	−0.11211	5.71825	−5.83362	−0.11701	5.71661	−5.84614	−0.12789	5.71825
menthol	−7.27230	−0.03837	7.23393	−7.03801	0.37089	7.40890	−7.04209	0.363554	7.40564	−7.04263	0.36219	7.40482

**Table 3:** Calculated GCRD for limonene and selected monoterpenes, structures optimized using the B3LYP/6-311+G method without and with PCM model.

	Terpene	*E*_0_ [a.u.]	I [eV]	A [eV]	X [eV]	η [eV]	S [eV]	ω [eV]	μ [eV]
	limonene	−390.76016952	6.419	0.024	3.221	3.197	0.156	1.623	−3.221
	cymene	−389.59360262	6.451	0.328	3.390	3.061	0.163	1.876	−3.390
	pinene	−390.74018343	6.207	−0.027	3.090	3.117	0.160	1.531	−3.090
	thymol	−464.83540277	6.087	0.392	3.239	2.847	0.176	1.843	−3.239
	menthol	−468.44884783	7.272	0.038	3.655	3.617	0.138	1.847	−3.655
MeCN	limonene	−390.76198136	6.144	−0.289	2.928	3.217	0.155	1.332	−2.928
	cymene	−389.59597035	6.230	0.118	3.174	3.056	0.164	1.648	−3.174
	pinene	−390.74104612	5.916	−0.327	2.794	3.121	0.160	1.251	−2.794
	thymol	−464.84156521	5.834	0.117	2.975	2.858	0.175	1.549	−2.975
	menthol	−468.45328899	7.042	−0.364	3.339	3.703	0.135	1.506	−3.339
H_2_O	limonene	−390.76203510	6.141	−0.293	2.923	3.217	0.155	1.328	−2.923
	cymene	−389.59604600	6.228	0.116	3.172	3.056	0.164	1.647	−3.172
	pinene	−390.74107098	5.911	−0.332	2.790	3.122	0.160	1.247	−2.790
	thymol	−464.84176291	5.830	0.112	2.971	2.859	0.175	1.544	−2.971
	menthol	−468.45341693	7.038	−0.371	3.334	3.705	0.135	1.500	−3.334
MeOH	limonene	−390.76197221	6.145	−0.288	2.929	3.216	0.155	1.333	−2.929
	cymene	−389.59595751	6.230	0.118	3.174	3.056	0.164	1.648	−3.174
	pinene	−390.74104188	5.916	−0.326	2.795	3.121	0.160	1.251	−2.795
	thymol	−464.84153739	5.846	0.128	2.987	2.859	0.175	1.560	−2.987
	menthol	−468.45326721	7.043	−0.362	3.340	3.702	0.135	1.507	−3.340

## Data Availability

The data presented in this study are available in the Supplementary Materials or on request from the corresponding author.

## Supplementary Materials

The following supporting information can be downloaded at: https://www.mdpi.com/article/10.3390/molecules29071579/s1, Table S1: Energy of the HOMO and LUMO orbitals along with ΔEgap levels for limonene and selected monoterpenes, structures optimized using different methods without the PCM model; Table S2: The energies (with and without zero point correction), enthalpies, free energies (G), and bound dissociation enthalpy (BDE) values for the limonene molecules and its radicals were calculated using B3LYP and water as the PCM model; Table S3: The energies (with and without zero point correction), enthalpies, free energies (G), and bound dissociation enthalpy (BDE) values for the cymene molecules and its radicals were calculated using B3LYP and water as the PCM model; Table S4: The energies (with and without zero point correction), enthalpies, free energies (G), and bound dissociation enthalpy (BDE) values for the pinene molecules and its radicals were calculated using B3LYP and water as the PCM model; Table S5: The energies (with and without zero point correction), enthalpies, free energies (G), and bound dissociation enthalpy (BDE) values for the thymol molecules and its radicals were calculated using B3LYP and water as the PCM model; Table S6: The energies (with and without zero point correction), enthalpies, free energies (G), and bound dissociation enthalpy (BDE) values for the menthol molecules and its radicals were calculated using B3LYP and water as the PCM model; Scheme S1: Possible oxidation products of selected monoterpenes.
