# Supplementary material for: DFT Studies of the Activity and Reactivity of Limonene in Comparison with Selected Monoterpenes
Source: Molecules. 2024 Apr 1;29(7):1579. doi: 10.3390/molecules29071579 (PMC11013946; doi:10.3390/molecules29071579)
Supplement: Supplementary file 1 [file molecules-29-01579-s001.zip › molecules-2926117-supplementary.pdf]

Supplementary Materials

DFT Studies of the Activity and Reactivity of Limonene in Comparison with Selected Monoterpenes

Katarzyna Rydel-Ciszek*

Department of Physical Chemistry, Faculty of Chemistry, Rzeszów University of Technology,

al. Powstańców Warszawy 6, 35–959 Rzeszów, Poland

** Correspondence: kasiar@prz.edu.pl (K.R.-C.)*

Table S1. Energy of the HOMO and LUMO orbitals along with ΔE_{gap} levels for limonene and selected monoterpenes, structures optimized using different methods without the PCM model.

	B3LYP/6-311+G(d)			B3PW91/6-311+G(d)			CAM-B3LYP/6-311+G(d)/ DEF2TZV			ωB97XD/6-311+G(d)/ DEF2TZV		
	E_{HOMO}, [eV]	E_{LUMO}, [eV]	E_{gap}, [eV]	E_{HOMO}, [eV]	E_{LUMO}, [eV]	E_{gap}, [eV]	E_{HOMO}, [eV]	E_{LUMO}, [eV]	E_{gap}, [eV]	E_{HOMO}, [eV]	E_{LUMO}, [eV]	E_{gap}, [eV]
limonene	−6.419	−0.024	6.395	−6.436	0.142	6.578	−7.928	0.780	8.708	−8.437	1.643	10.079
cymene	−6.451	−0.328	6.123	−6.492	−0.304	6.188	−7.855	0.806	8.661	−8.392	1.590	9.982
pinene	−6.207	0.027	6.235	−6.211	0.270	6.482	−7.718	0.807	8.525	−8.224	1.678	9.902
thymol	−6.087	−0.392	5.695	−6.103	−0.352	5.750	−7.494	0.625	8.118	−8.013	1.507	9.520
menthol	−7.272	−0.038	7.234	−7.232	0.261	7.493	−8.944	0.752	9.697	−9.393	1.631	11.025

Table S2. The energies (with and without zero point correction), enthalpies, free energies (G), and bound dissociation enthalpy (BDE) values for the limonene molecules and its radicals were calculated using B3LYP and water as the PCM model.

	**Molecules**	**Electronic Energy** [a.u.] ε_0	Sum of electronic and zero–point **Energies** [a.u.] ε_0+ZPE	Sum of electronic and thermal **Energies** [a.u.] ε_0+E_{tot}	Sum of electronic and thermal **Enthalpies** [a.u.] ε_0+H_{corr}	Sum of electronic and thermal **Free Energies** [a.u.] ε_0+G_{corr}	BDE [kcal/mol]
**6–31g(d)**	Limonene	−390.723274	−390.487083	−390.476384	−390.475440	−390.522996	–
	Limonene bond C**3**–H	−390.083064	−389.860782	−389.850005	−389.849060	−389.897609	80.61
	Limonene bond C**4**–H	−390.081014	−389.858711	−389.847983	−389.847039	−389.895407	81.88
	Limonene bond C**6**–H	−390.079414	−389.857634	−389.846560	−389.845616	−389.895805	82.77
	Limonene bond C**7**–H	−390.074327	−389.851753	−389.841326	−389.840382	−389.888014	86.05
	Limonene bond C**10**–H	−390.074069	−389.851785	−389.841332	−389.840387	−389.888063	86.05
	Limonene bond C**5**–H	−390.055788	−389.834694	−389.823626	−389.822682	−389.871925	97.16
	Limonene bond C**2**–H	−390.040630	−389.817306	−389.806558	−389.805614	−389.853932	107.87
	H atom	−0.500281	−0.500281	−0.498865	−0.497921	−0.510936	–
**Def2SVP**	Limonene	−390.442898	−390.209224	−390.198477	−390.197533	−390.245133	–
	Limonene bond C**3**–H	−389.803888	−389.583857	−389.573045	−389.572101	−389.620704	79.85
	Limonene bond C**4**–H	−389.801440	−389.581636	−389.570826	−389.569882	−389.618403	81.24
	Limonene bond C**6**–H	−389.800006	−389.580607	−389.569415	−389.568471	−389.619925	82.12
	Limonene bond C**7**–H	−389.795201	−389.574792	−389.564335	−389.563391	−389.611020	85.31
	Limonene bond C**10**–H	−389.794682	−389.574604	−389.564095	−389.56315	−389.611017	85.46
	Limonene bond C**5**–H	−389.776801	−389.558078	−389.546954	−389.54601	−389.595370	96.22
	Limonene bond C**2**–H	−389.760030	−389.539095	−389.528307	−389.527363	−389.575713	107.92
	H atom	−0.500549	−0.50055	−0.499134	−0.498189	−0.511204	–

Table S3. The energies (with and without zero point correction), enthalpies, free energies (G), and bound dissociation enthalpy (BDE) values for the cymene molecules and its radicals were calculated using B3LYP and water as the PCM model.

	**Molecules**	**Electronic Energy** [a.u.] ε_0	Sum of electronic and zero–point **Energies** [a.u.] ε_0+ZPE	Sum of electronic and thermal **Energies** [a.u.] ε_0+E*tot*	Sum of electronic and thermal **Enthalpies** [a.u.] ε_0+H*corr*	Sum of electronic and thermal **Free Energies** [a.u.] ε_0+G*corr*	BDE [kcal/mol]
**6–31g(d)**	Cymene	–389.555152	–389.341949	–389.331436	–389.330491	–389.378865	–
	Cymene bond C**8**–H	–388.910371	–388.711413	–388.700480	–388.699536	–388.749801	83.48
	Cymene bond C**7**–H	–388.903557	–388.703594	–388.693642	–388.692698	–388.739302	87.77
	Cymene bond C**9**–H	–388.881739	–388.683809	–388.672956	–388.672012	–388.721604	100.75
	Cymene bond C**10**–H	–388.881739	–388.683818	–388.672965	–388.672021	–388.721530	100.75
	Cymene bond C**2**–H	–388.867216	–388.666970	–388.656483	–388.655539	–388.704164	111.09
	Cymene bond C**6**–H	–388.867103	–388.666823	–388.656324	–388.655380	–388.704203	111.19
	Cymene bond C**3**–H	–388.866994	–388.666771	–388.656256	–388.655312	–388.704222	111.23
	Cymene bond C**5**–H	–388.866989	–388.666775	–388.666775	–388.655310	–388.704228	111.23
**Def2SVP**	Cymene	–389.275316	–389.063989	–389.053455	–389.052511	–389.101032	–
	Cymene bond C**8**–H	–388.631996	–388.434799	–388.424782	–388.423838	–388.470953	81.88
	Cymene bond C**7**–H	–388.624933	–388.426488	–388.416528	–388.415584	–388.462345	87.06
	Cymene bond C**9**–H	–388.603421	–388.407084	–388.396206	–388.395262	–388.445269	99.81
	Cymene bond C**10**–H	–388.601681	–388.405119	–388.394211	–388.393267	–388.443655	101.06
	Cymene bond C**2**–H	–388.587080	–388.388728	–388.378208	–388.377263	–388.426110	111.11
	Cymene bond C**6**–H	–388.586948	–388.388562	–388.378030	–388.377086	–388.426118	111.22
	Cymene bond C**3**–H	–388.587821	–388.388531	–388.377978	–388.377978	–388.426325	110.66
	Cymene bond C**5**–H	–388.586875	–388.388562	–388.377975	–388.377031	–388.426890	111.25

Table S4. The energies (with and without zero point correction), enthalpies, free energies (G), and bound dissociation enthalpy (BDE) values for the pinene molecules and its radicals were calculated using B3LYP and water as the PCM model.

	**Molecules**	**Electronic Energy** [a.u.] ε_0	Sum of electronic and zero–point **Energies** [a.u.] ε_0+ZPE	Sum of electronic and thermal **Energies** [a.u.] ε_0+E_{tot}	Sum of electronic and thermal **Enthalpies** [a.u.] ε_0+H_{corr}	Sum of electronic and thermal **Free Energies** [a.u.] ε_0+G_{corr}	BDE [kcal/mol]
**6–31g(d)**	Pinene	–390.708843	–390.471941	–390.462154	–390.461210	–390.505616	–
	Pinene bond C**8**–H	–390.079188	–389.856260	–389.845401	–389.845401	–389.893485	73.98
	Pinene bond C**5**–H	–390.065830	–389.844644	–389.833299	–389.832355	–389.882267	82.16
	Pinene bond C**10**–H	–390.064658	–389.841275	–389.831792	–389.830848	–389.875179	83.11
	Pinene bond C**7**–H	–390.056664	–389.835537	–389.824540	–389.823596	–389.872224	87.66
	Pinene bond C**6**–H	–390.035119	–389.811436	–389.801625	–389.800681	–389.800681	102.04
	Pinene bond C**9**–H	–390.033759	–389.811450	–389.801417	–389.800473	–389.845898	102.17
	Pinene bond C**2**–H	–390.032536	–389.808942	–389.799074	–389.798130	–389.843390	103.64
	Pinene bond C**4**–H	–390.023077	–389.798840	–389.789024	–389.788079	–389.833281	109.95
**Def2SVP**	Pinene	–390.428322	–390.193871	–390.184076	–390.183132	–390.227508	–
	Pinene bond C**8**–H	–389.798736	–389.579128	–389.568160	–389.567216	–389.617346	73.87
	Pinene bond C**5**–H	–389.787027	–389.568127	–389.556749	–389.555805	–389.605826	81.04
	Pinene bond C**10**–H	–389.785459	–389.564175	–389.554702	–389.553757	–389.598022	82.32
	Pinene bond C**7**–H	–389.777872	–389.559032	–389.548007	–389.547063	–389.595758	86.52
	Pinene bond C**6**–H	–389.754627	–389.533279	–389.523462	–389.522518	–389.567580	101.92
	Pinene bond C**9**–H	–389.754602	–389.534857	–389.524639	–389.523695	–389.569475	101.18
	Pinene bond C**2**–H	–389.752097	–389.530830	–389.520950	–389.520006	–389.565246	103.50
	Pinene bond C**4**–H	–389.742248	–389.520352	–389.510525	–389.509581	–389.554772	110.04

Table S5. The energies (with and without zero point correction), enthalpies, free energies (G), and bound dissociation enthalpy (BDE) values for the thymol molecules and its radicals were calculated using B3LYP and water as the PCM model.

	**Molecules**	**Electronic Energy** [a.u.] ε_0	Sum of electronic and zero–point **Energies** [a.u.] ε_0+ZPE	Sum of electronic and thermal **Energies** [a.u.] ε_0+E*tot*	Sum of electronic and thermal **Enthalpies** [a.u.] ε_0+H*corr*	Sum of electronic and thermal **Free Energies** [a.u.] ε_0+G*corr*	BDE [kcal/mol]
**6–31g(d)**	Thymol	–464.776596	–464.559153	–464.547553	–464.546609	–464.597260	–
	Thymol bond O–H	–464.143389	–463.938570	–463.927308	–463.926364	–463.976194	76.76
	Thymol bond C**7**–H	–464.127874	–463.924706	–463.912660	–463.911716	–463.964917	85.95
	Thymol bond C**10**–H	–464.124821	–463.920626	–463.909603	–463.908659	–463.957230	87.87
	Thymol bond C**9**–H	–464.103276	–463.901672	–463.890244	–463.889300	–463.940314	100.02
	Thymol bond C**8**–H	–464.102683	–463.900181	–463.888245	–463.887301	–463.887301	101.27
	Thymol bond C**2**–H	–464.089812	–463.885584	–463.873891	–463.872946	–463.924230	110.28
	Thymol bond C**3**–H	–464.086981	–463.882450	–463.870837	–463.869893	–463.921101	112.20
	Thymol bond C**5**–H	–464.086987	–463.882211	–463.870657	–463.869713	–463.920262	112.31
**Def2SVP**	Thymol	–464.446307	–464.230377	–464.218875	–464.217930	–464.267764	–
	Thymol bond O–H	–463.806235	–463.603106	–463.591876	–463.590932	–463.640595	80.83
	Thymol bond C**7**–H	–463.798723	–463.596914	–463.585924	–463.584980	–463.634279	84.56
	Thymol bond C**10**–H	–463.795752	–463.592817	–463.581838	–463.580894	–463.629384	87.13
	Thymol bond C**9**–H	–463.774592	–463.573671	–463.561814	–463.560870	–463.612099	99.69
	Thymol bond C**8**–H	–463.773767	–463.572579	–463.560709	–463.559765	–463.611098	100.39
	Thymol bond C**2**–H	–463.759118	–463.556407	–463.544787	–463.543842	–463.594885	110.38
	Thymol bond C**3**–H	–463.756372	–463.553505	–463.541904	–463.540960	–463.592329	112.19
	Thymol bond C**5**–H	–463.756234	–463.553025	–463.541494	–463.540549	–463.591022	112.44

Table S6. The energies (with and without zero point correction), enthalpies, free energies (G), and bound dissociation enthalpy (BDE) values for the menthol molecules and its radicals were calculated using B3LYP and water as the PCM model.

	**Molecules**	**Electronic Energy** [a.u.] ε_0	Sum of electronic and zero–point **Energies** [a.u.] ε_0+ZPE	Sum of electronic and thermal **Energies** [a.u.] ε_0+E_{tot}	Sum of electronic and thermal **Enthalpies** [a.u.] ε_0+H_{corr}	Sum of electronic and thermal **Free Energies** [a.u.] ε_0+G_{corr}	BDE [kcal/mol]
**6–31g(d)**	Menthol	–468.398657	–468.109603	–468.097407	–468.096463	–468.146508	–
	Menthol bond C**6**–H	–467.741171	–467.465332	–467.452765	–467.452765	–467.503732	91.48
	Menthol bond C**4**–H	–467.737287	–467.462931	–467.450209	–467.449265	–467.501841	93.67
	Menthol bond C**1**–H	–467.736156	–467.461144	–467.448672	–467.447728	–467.499759	94.64
	Menthol bond O–H	–467.734646	–467.459417	–467.447360	–467.446416	–467.446416	95.46
	Menthol bond C**2**–H	–467.732684	–467.458864	–467.446221	–467.445277	–467.497167	96.18
	Menthol bond C**3**–H	–467.731106	–467.456837	–467.444297	–467.443353	–467.494840	97.38
	Menthol bond C**5**–H	–467.730480	–467.455994	–467.443530	–467.442586	–467.494084	97.86
	Menthol bond C**8**–H	–467.728494	–467.454422	–467.442182	–467.441238	–467.491958	98.71
	Menthol bond C**10**–H	–467.726818	–467.453073	–467.440548	–467.439604	–467.490944	99.74
	Menthol bond C**9**–H	–467.725786	–467.451985	–467.439359	–467.438415	–467.490087	100.48
**Def2SVP**	Menthol	–468.064812	–467.778318	–467.766109	–467.765164	–467.815239	–
	Menthol bond C**6**–H	–467.409890	–467.137290	–467.124904	–467.123960	–467.175183	89.74
	Menthol bond C**4**–H	–467.404233	–467.132427	–467.119683	–467.118739	–467.171407	93.02
	Menthol bond C**1**–H	–467.403011	–467.130489	–467.118025	–467.11708	–467.168926	94.06
	Menthol bond O–H	–467.394396	–467.121912	–467.109829	–467.108885	–467.159674	99.20
	Menthol bond C**2**–H	–467.399940	–467.128458	–467.115860	–467.114915	–467.166685	95.42
	Menthol bond C**3**–H	–467.398295	–467.126447	–467.113891	–467.112947	–467.164486	96.65
	Menthol bond C**5**–H	–467.397400	–467.125294	–467.112832	–467.111888	–467.163345	97.32
	Menthol bond C**8**–H	–467.393982	–467.122686	–467.109984	–467.109040	–467.160725	99.11
	Menthol bond C**10**–H	–467.394365	–467.122858	–467.110359	–467.109415	–467.160694	98.87
	Menthol bond C**9**–H	–467.393240	–467.121687	–467.109065	–467.108121	–467.159792	99.68

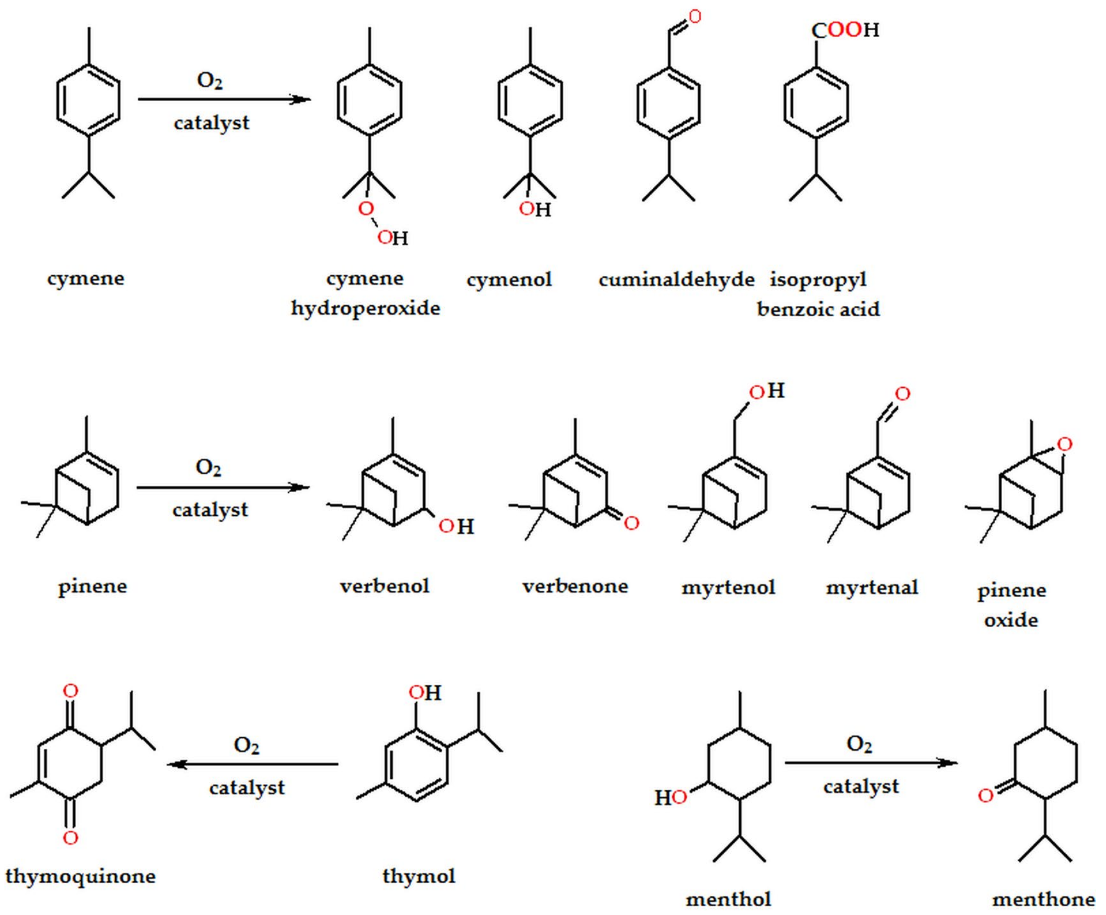

Scheme S1. Possible oxidation products of selected monoterpenes.
